# mHealth and telemedicine apps: in search of a common regulation

**DOI:** 10.3332/ecancer.2018.853

**Published:** 2018-07-11

**Authors:** Chiara Crico, Chiara Renzi, Norbert Graf, Alena Buyx, Haridimos Kondylakis, Lefteris Koumakis, Gabriella Pravettoni

**Affiliations:** 1 Department of Oncology and Hemato-Oncology, University of Milan, Milan, Italy; 2 Applied Research Division for Cognitive and Psychological Science, European Institute of Oncology, Milan 20141, Italy; 3 Department of Pediatric Oncology and Hematology, Saarland University, Homburg/Saar, Germany; 4 Department of Biomedical Ethics, Institute of Experimental Medicine, University of Kiel, Germany; 5 Computational Biomedicine Laboratory, FORTH-ICS, Heraklion, Greece

**Keywords:** telemedicine, data protection, patient data privacy, mobile apps, European community

## Abstract

Developments in information and communication technology have changed the way healthcare processes are experienced by both patients and healthcare professionals: more and more services are now available through computers and mobile devices. Smartphones are becoming useful tools for managing one’s health, and today, there are many available apps meant to increase self-management, empowerment and quality of life. However, there are concerns about the implications of using mHealth and apps: data protection issues, concerns about sharing information online, and the patients’ capacity for discerning effective and valid apps from useless ones. The new General Data Protection Regulation has been introduced in order to give uniformity to data protection regulations among European countries but shared guidelines for mHealth are yet to develop. A unified perspective across Europe would increase the control over mHealth exploitation, making it possible to think of mHealth as effective and standard tools for future medical practice.

## Introduction

Over the last two decades, information and communication technology (ICT) innovations have reached the field of health and healthcare, empowering citizens to take care of their own health, providing resources for patients and healthcare professionals, and improving the efficacy of service provision by hospitals and healthcare systems [1, 2].

The introduction of mobile technologies represents a substantial change in the way individuals relate to medicine and healthcare. For instance, 52% of smartphone owners use their device to search the Internet for health-related information [4]; smartphones and tablets have become the preferred instruments for healthcare practitioners who need to find information from their workplace [4].

Electronic Health (eHealth) can be defined as the safe and productive use of ICT in support of healthcare professionals and patients in health-related fields [1]. Its specific aim is to help and support all healthcare processes, ranging from prevention to detecting health problems, from diagnosis to disease treatment. eHealth may be used to support both professionals and patients: it may provide services for patients, such as monitoring at a distance, remote diagnosis, consultation, home care and training with self-care management, thus empowering patients relative to their disease [5]. At the same time, eHealth can decrease the workload of healthcare professionals, e.g. simplifying inter-professional communication, by providing an easy way to share information involving patients sharing common problems, as well as education and training from a distance [2, 6, 7].

These services are available even via mobile and wireless technologies: Mobile Health (mHealth) represents a subset of eHealth, namely the application of mobile technology to provide or use health services, share clinical information and collect data [7, 8]. mHealth offers the possibility to have a fast diagnosis, to provide a feedback system in order to monitor health status, promoting healthy behaviour and encouraging changes to dysfunctional behaviours; to provide easy access to treatment and rehabilitation; to receive electronic prescriptions or obtain informed consent rapidly, thereby cutting waiting times [1, 9]. Some apps work as information providers, improving health consciousness and literacy, as they give users an easy and portable access to educational material [7, 10–13].

Due to the innovative opportunities they offer, mHealth is rapidly spreading in clinical contexts [14]. One of these is the practice of telemedicine, which is the use of ICT to deliver healthcare services by healthcare professionals; it allows a safe exchange of information, enabling people to communicate health-related issues—such as prevention, diagnosis, treatment and follow-up—from a distance, overcoming logistic and long distance criticalities [15].

Nowadays, more than 165,000 apps related to health behaviour are available in online stores. The most common are those related to fitness, behaviour and nutrition [16]. Wellness applications are designed to help people in embracing and maintaining a healthy behaviour, as they deliver interventions that can be customised and may encourage users’ adherence through an interactive system of messages and feedback [3, 17]. With the promises, ‘this app will help you to lose weight’, ‘this app will lower your blood pressure’, apps promoting fitness and healthy lifestyles are gaining success and their use is associated with the intention to change nutrition behaviour and improve physical activity [18]. Due to the nature of the information these apps require, there are issues of data protection and data flow to consider [21], as well as commercial interests in data collected by mHealth apps [22]. In addition, it has also been shown that health apps do not always provide entirely accurate health information to patients [19] and worries exist about their appropriate validation [20]: it should be considered that these apps and their functions are usually tested for usability rather than for medical efficacy. In order to overcome this issue, the Horizon 2020 European project iManageCancer developed an online platform with the aim of empowering cancer patients and strengthening self-management in cancer diseases, and designed a pilot study to test the platform’s efficacy.

## Discussion

Within the broad range of medical apps, some have been specifically designed for patients affected by illnesses with long trajectories. Some mHealth apps are meant for chronic sufferers and cancer patients, to help them manage their different therapies, monitor their symptoms, and improve their adherence to therapy; such tools facilitate patient-physician communication and increase the possibility of remote control, providing real-time exchange of information [23–25].

Consequently, these tools attracted the attention of regulatory organisms in the United States and in Europe. Title II of the Health Insurance Portability and Accountability Act, enacted by the United States Congress in 1996, introduces policies to protect data privacy and the security of individuals’ medical information [26]. Then in 2009, the United States Department of Health and Human Services released the Health Information Technology for Economic and Clinical Health Act, a legislation act meant to promote health information technology [27]. In 2015, the Food and Drug Administration established that mHealth apps can fall under the definition of a medical device, when ‘the intended use of a mobile app is for the diagnosis of disease or other conditions, or the cure, mitigation, treatment, or prevention of disease, or is intended to affect the structure or any function of the body of man’ [28]. For the European Commission, the definition of medical device applies to any kind of instrument, including software, which are used for: ‘diagnosis, prevention, monitoring, treatment or alleviation of a disease or a handicap; investigation, replacement or modification of the anatomy or of a physiological process; control of conception’ [29].

In order to promote eHealth and mHealth, in 2012 the European Council released the eHealth Action Plan 2012–2020, the first formal commitment from all Member States to cooperate in the field of eHealth. The goals of the Action Plan range from the creation of an eHealth record to the online set up of health services, such as information on healthy behaviour and prevention of illnesses. Nevertheless, the lack of a uniform regulation among the countries remains a problem and the eHealth Action Plan points it out, underlining the importance of ‘promoting synergies between related policies and stakeholders, so as to develop better solutions, prevent market fragmentation and disseminate best practices’ [31].

The European Union has no uniform regulations for what concerns healthcare and medicine in general; the Directive 2011/24/EU provides the only guidelines available about health services, but many issues are not covered by any European regulation, especially what concerns medical liability [32]. Due to the differences in penal laws among European countries, it is hard to imagine a shared set of norms concerning eHealth, since the lack of uniformity potentially compromises its development [32]. The only directives concerning eHealth are the regulations on information service and data protection: Directive 95/46/EU, 200/31/EC and 2002/58/EC, specifically on ICT [32] and the new General Data Protection Regulation (GDPR) that will enter into force in May 2018.

The new GDPR has been introduced to harmonise privacy and data protection legislation within the European countries. Before the GDPR, most of the Member States had no specific laws on eHealth: each country asserted the general laws on data protection and professional conduct.

## Conclusion

Developing novel regulation is made complex by the fact that it has to appropriately address a number of ethical issues relating to health apps. These include the aforementioned problems around accuracy and validity of data as well as data security and fair data flow, but also challenges of understanding and interpretation when patients have to make sense of health information on their own [33]. In addition and particularly considering mHealth instruments that have been designed for patients, it has to be discussed whether or not to require clinicians to treat patients initially in person before accessing, e.g. teleservices [34].

Regarding apps, the European Commission released a Working Document assessing some generic regulations for apps falling under the definition of a medical device [35], but the European legal framework is not sufficiently adapted yet to the regulatory needs arising from mHealth.

First, health apps seem promising tools to increase self-management, empowerment and quality of life [36], and studies involving smartphone apps are increasing in number. However, the efficacy of these tools is difficult to assess and the correlation between apps usage and positive health outcomes is still under investigation [37]. Due to the relatively recent emergence of this technology, evidence-based understanding of its effects has to be improved and further research on this topic is needed. It should also be considered that building a controlled research project may be complex because the testing of apps often implies patients using them in their everyday life; what emerged from the pilot study conducted for the project iManageCancer is the difficulty of combining everyday life with the many functions of the platform. Contrasting results on the effectiveness of these apps may thus depend on how these tools are used: an uncontrolled path entails many challenges in terms of adherence and data collection [38] and this problem is not easily overcome. However, efficacy remains a very important aim to reach, in order to meet patients’ needs.

Furthermore, it should be noted that the use of health apps is not free from risks. In general, health apps are not considered particularly dangerous; however, there is a series of acknowledged limitations and risks: one of them relates to the exposure of individuals’ privacy and security [18, 39]. When using web-based apps, private and sensitive data are collected, linked and analysed, including when data are transmitted from patient to physician [33]. Developing and implementing data protection that is in line with existing European and national regulation, and at the same time appropriately applicable to mHealth, is one of the current challenges for societies and changing medical landscapes [40–42].

Another risk concerns the use of information provided by the apps, as mentioned briefly above [1]: many of the health apps act as information channels, but how can we be sure that people understand this information properly? Health literacy varies significantly between populations and abilities as well as cultural aspects shaping understanding of health information and subsequent behaviour differ [43]. The concern about patients’ (and citizens’, in general) literacy and the risk of inaccurate or misleading information is highly relevant [44]: mobile applications are not necessarily based on accepted scientific data and the lack of research and validity of these apps poses a question on who is accountable in case something goes wrong [1]. Concerning apps, generally, the producer is considered liable for damages caused by its product by Council Directive 85/374/EEC [6]; hence, consumer protection applies once the app is released. However, unless an app has been investigated as a medical device, there is no control *a priori* over its content. The concept of efficacy is still rather unusual for app developers: the success of an app is measured by the number of downloads and ratings, rather than effectiveness [38]. This might change, depending on if health apps will be classed more consistently as medical devices. Currently, however, this is a developing issue. For what concerns the iManageCancer project, designing the platform the developers based the apps on the scientific literature, with the aim of giving the patients verified information and reliable functions to manage their illness.

The incoming GDPR is implemented with some degree of difference between countries. The lack of a shared understanding of the legislation in Europe and the different interpretations it allows, and even more so, the lack of specific guidelines for mHealth guidelines make the use of mHealth tools challenging. They have great potential and could become effective and standard tools for future medical practice [41]. However, to avoid the practical and ethical issues mentioned above, a unified perspective across Europe and clear and specific guidelines both on clinical trials to test apps’ efficacy as well as on their use in clinical practice would allow more effective control over the use of telemedicine and more specific apps. This, in turn, would make it possible to ascertain the quality and validity of a particular instrument’s information, issues of data protection, etc., and how it is being used remotely, with the foreseeable result of increasing its appeal and ultimately, its adoption [30].
